# Supplementary material for: Modeling glioblastoma heterogeneity as a dynamic network of cell states
Source: Mol Syst Biol. 2021 Sep 16;17(9):e10105. doi: 10.15252/msb.202010105 (PMC8444284; doi:10.15252/msb.202010105)
Supplement: Supplementary file 5 — Source Data for Figure 3 [file MSB-17-e10105-s001.zip › Figure3A_sourcedata/GSEA_3065/nosigdb_state5.GseaPreranked.1622718435914/RICHARDS_NOWAKOWSKI_SCIENCE_2017_IPC_NEN3_UPREG.html]

Details for gene set RICHARDS\_NOWAKOWSKI\_SCIENCE\_2017\_IPC\_NEN3\_UPREG[GSEA]

|  || Dataset | state5 |
| Phenotype | NoPhenotypeAvailable |
| Upregulated in class | na\_neg |
| GeneSet | RICHARDS\_NOWAKOWSKI\_SCIENCE\_2017\_IPC\_NEN3\_UPREG |
| Enrichment Score (ES) | -0.53155816 |
| Normalized Enrichment Score (NES) | -2.0124912 |
| Nominal p-value | 0.0 |
| FDR q-value | 0.0 |
| FWER p-Value | 0.0 |
Table: GSEA Results Summary

  

Fig 1: Enrichment plot: RICHARDS\_NOWAKOWSKI\_SCIENCE\_2017\_IPC\_NEN3\_UPREG      
 Profile of the Running ES Score & Positions of GeneSet Members on the Rank Ordered List

  

| PROBE | GENE SYMBOL | GENE\_TITLE | RANK IN GENE LIST | RANK METRIC SCORE | RUNNING ES | CORE ENRICHMENT || 1 | CD63 |  |  | 24 | 0.769 | 0.0086 | No |
| 2 | LAPTM4A |  |  | 51 | 0.641 | 0.0150 | No |
| 3 | SLC1A3 |  |  | 82 | 0.550 | 0.0197 | No |
| 4 | PON2 |  |  | 84 | 0.546 | 0.0276 | No |
| 5 | MSMO1 |  |  | 92 | 0.540 | 0.0347 | No |
| 6 | DLL3 |  |  | 171 | 0.463 | 0.0326 | No |
| 7 | PNN |  |  | 195 | 0.442 | 0.0365 | No |
| 8 | MAGI1 |  |  | 196 | 0.442 | 0.0430 | No |
| 9 | PTTG1IP |  |  | 214 | 0.430 | 0.0473 | No |
| 10 | RPN2 |  |  | 244 | 0.411 | 0.0501 | No |
| 11 | SEC11A |  |  | 247 | 0.411 | 0.0559 | No |
| 12 | SLC4A7 |  |  | 250 | 0.409 | 0.0617 | No |
| 13 | TMX1 |  |  | 255 | 0.406 | 0.0672 | No |
| 14 | CHD7 |  |  | 312 | 0.376 | 0.0663 | No |
| 15 | GPC2 |  |  | 367 | 0.354 | 0.0653 | No |
| 16 | FJX1 |  |  | 371 | 0.352 | 0.0702 | No |
| 17 | PAXBP1 |  |  | 392 | 0.344 | 0.0729 | No |
| 18 | GON4L |  |  | 397 | 0.342 | 0.0775 | No |
| 19 | SEZ6L |  |  | 422 | 0.334 | 0.0797 | No |
| 20 | CACNA2D1 |  |  | 454 | 0.325 | 0.0809 | No |
| 21 | AASS |  |  | 494 | 0.313 | 0.0811 | No |
| 22 | GJC1 |  |  | 588 | 0.288 | 0.0747 | No |
| 23 | MLC1 |  |  | 591 | 0.288 | 0.0787 | No |
| 24 | HES6 |  |  | 653 | 0.274 | 0.0757 | No |
| 25 | ZNF292 |  |  | 656 | 0.274 | 0.0795 | No |
| 26 | ZKSCAN1 |  |  | 678 | 0.269 | 0.0811 | No |
| 27 | CELSR2 |  |  | 750 | 0.251 | 0.0767 | No |
| 28 | GPRC5B |  |  | 838 | 0.234 | 0.0702 | No |
| 29 | ADAR |  |  | 857 | 0.232 | 0.0715 | No |
| 30 | PGRMC2 |  |  | 950 | 0.219 | 0.0642 | No |
| 31 | BAZ2B |  |  | 974 | 0.214 | 0.0647 | No |
| 32 | LRP8 |  |  | 1029 | 0.207 | 0.0616 | No |
| 33 | MEGF10 |  |  | 1049 | 0.204 | 0.0624 | No |
| 34 | C1orf61 |  |  | 1057 | 0.204 | 0.0646 | No |
| 35 | CELSR1 |  |  | 1257 | 0.178 | 0.0445 | No |
| 36 | ZNF253 |  |  | 1283 | 0.174 | 0.0442 | No |
| 37 | CLASRP |  |  | 1367 | 0.164 | 0.0372 | No |
| 38 | POU2F1 |  |  | 1467 | 0.156 | 0.0281 | No |
| 39 | POU3F2 |  |  | 1559 | 0.146 | 0.0199 | No |
| 40 | CBFA2T2 |  |  | 1561 | 0.145 | 0.0219 | No |
| 41 | SETBP1 |  |  | 1570 | 0.144 | 0.0231 | No |
| 42 | TMEM98 |  |  | 1598 | 0.141 | 0.0221 | No |
| 43 | IST1 |  |  | 1635 | 0.139 | 0.0200 | No |
| 44 | DPF2 |  |  | 1648 | 0.138 | 0.0207 | No |
| 45 | UBE2O |  |  | 1673 | 0.135 | 0.0199 | No |
| 46 | ZFHX4 |  |  | 1700 | 0.133 | 0.0189 | No |
| 47 | NKAIN3 |  |  | 1766 | 0.129 | 0.0134 | No |
| 48 | ZNF462 |  |  | 1814 | 0.126 | 0.0099 | No |
| 49 | RFTN2 |  |  | 1867 | 0.121 | 0.0057 | No |
| 50 | SRRM2 |  |  | 1935 | 0.117 | -0.0002 | No |
| 51 | FLNB |  |  | 2024 | 0.110 | -0.0087 | No |
| 52 | LIMD1 |  |  | 2036 | 0.109 | -0.0083 | No |
| 53 | TMTC2 |  |  | 2057 | 0.108 | -0.0090 | No |
| 54 | DAAM1 |  |  | 2069 | 0.107 | -0.0087 | No |
| 55 | BTG2 |  |  | 2174 | 0.100 | -0.0191 | No |
| 56 | FRMD4A |  |  | 2196 | 0.098 | -0.0201 | No |
| 57 | AGO1 |  |  | 2254 | 0.094 | -0.0252 | No |
| 58 | ZHX3 |  |  | 2255 | 0.094 | -0.0238 | No |
| 59 | EGR1 |  |  | 2336 | 0.090 | -0.0316 | No |
| 60 | MED12L |  |  | 2363 | 0.088 | -0.0333 | No |
| 61 | IVNS1ABP |  |  | 2393 | 0.086 | -0.0353 | No |
| 62 | DENND1B |  |  | 2448 | 0.082 | -0.0403 | No |
| 63 | MDK |  |  | 2458 | 0.082 | -0.0401 | No |
| 64 | MED27 |  |  | 2716 | 0.067 | -0.0685 | No |
| 65 | ARMCX6 |  |  | 2842 | 0.061 | -0.0819 | No |
| 66 | SYNE2 |  |  | 2881 | 0.058 | -0.0854 | No |
| 67 | CA12 |  |  | 2885 | 0.058 | -0.0848 | No |
| 68 | SCHIP1 |  |  | 2911 | 0.057 | -0.0869 | No |
| 69 | MEX3A |  |  | 2917 | 0.057 | -0.0866 | No |
| 70 | COL11A1 |  |  | 2950 | 0.055 | -0.0894 | No |
| 71 | HEG1 |  |  | 3009 | 0.051 | -0.0953 | No |
| 72 | PTPRS |  |  | 3026 | 0.050 | -0.0964 | No |
| 73 | HDAC9 |  |  | 3051 | 0.049 | -0.0984 | No |
| 74 | SRSF7 |  |  | 3126 | 0.046 | -0.1062 | No |
| 75 | SYNRG |  |  | 3139 | 0.045 | -0.1069 | No |
| 76 | CORO1C |  |  | 3217 | 0.040 | -0.1151 | No |
| 77 | ACTR6 |  |  | 3220 | 0.040 | -0.1147 | No |
| 78 | TFDP2 |  |  | 3271 | 0.038 | -0.1199 | No |
| 79 | SLC22A23 |  |  | 3276 | 0.038 | -0.1198 | No |
| 80 | TARS2 |  |  | 3283 | 0.037 | -0.1199 | No |
| 81 | FOXN2 |  |  | 3403 | 0.032 | -0.1331 | No |
| 82 | PSME4 |  |  | 3445 | 0.030 | -0.1373 | No |
| 83 | CACUL1 |  |  | 3466 | 0.028 | -0.1392 | No |
| 84 | ARID1A |  |  | 3598 | 0.023 | -0.1538 | No |
| 85 | USP3 |  |  | 3650 | 0.022 | -0.1593 | No |
| 86 | FANCF |  |  | 3768 | 0.017 | -0.1724 | No |
| 87 | FGD4 |  |  | 3777 | 0.016 | -0.1731 | No |
| 88 | PAK3 |  |  | 3876 | 0.013 | -0.1841 | No |
| 89 | MAP6 |  |  | 3889 | 0.012 | -0.1853 | No |
| 90 | PPME1 |  |  | 3965 | 0.008 | -0.1937 | No |
| 91 | RGL1 |  |  | 4007 | 0.007 | -0.1983 | No |
| 92 | TMEM258 |  |  | 4058 | 0.005 | -0.2039 | No |
| 93 | IRGQ |  |  | 4253 | -0.003 | -0.2261 | No |
| 94 | CDC37L1 |  |  | 4312 | -0.006 | -0.2326 | No |
| 95 | CCND2 |  |  | 4539 | -0.016 | -0.2582 | No |
| 96 | ZNF232 |  |  | 4581 | -0.017 | -0.2626 | No |
| 97 | SDC2 |  |  | 4596 | -0.018 | -0.2639 | No |
| 98 | PSMD5 |  |  | 4655 | -0.020 | -0.2703 | No |
| 99 | KAT6A |  |  | 4783 | -0.025 | -0.2844 | No |
| 100 | NOL4L |  |  | 4897 | -0.029 | -0.2969 | No |
| 101 | SVIL |  |  | 4953 | -0.031 | -0.3027 | No |
| 102 | MDM2 |  |  | 4954 | -0.031 | -0.3022 | No |
| 103 | GTF2I |  |  | 4961 | -0.032 | -0.3025 | No |
| 104 | INSM1 |  |  | 5071 | -0.036 | -0.3144 | No |
| 105 | STAM |  |  | 5090 | -0.037 | -0.3159 | No |
| 106 | MYO10 |  |  | 5109 | -0.037 | -0.3174 | No |
| 107 | CTTN |  |  | 5123 | -0.038 | -0.3183 | No |
| 108 | DUSP12 |  |  | 5226 | -0.041 | -0.3294 | No |
| 109 | TP53 |  |  | 5275 | -0.043 | -0.3342 | No |
| 110 | TCF3 |  |  | 5402 | -0.048 | -0.3479 | No |
| 111 | RBPJ |  |  | 5474 | -0.051 | -0.3553 | No |
| 112 | KIF26B |  |  | 5534 | -0.054 | -0.3612 | No |
| 113 | IFT74 |  |  | 5565 | -0.056 | -0.3638 | No |
| 114 | OXR1 |  |  | 5592 | -0.057 | -0.3659 | No |
| 115 | GOLGA4 |  |  | 5615 | -0.058 | -0.3676 | No |
| 116 | RRP8 |  |  | 5699 | -0.062 | -0.3762 | No |
| 117 | CHMP1B |  |  | 5886 | -0.069 | -0.3964 | No |
| 118 | NCOA5 |  |  | 5964 | -0.072 | -0.4041 | No |
| 119 | BAZ1A |  |  | 6008 | -0.074 | -0.4079 | No |
| 120 | TFAP2C |  |  | 6012 | -0.075 | -0.4072 | No |
| 121 | JAKMIP2 |  |  | 6179 | -0.083 | -0.4249 | No |
| 122 | QSER1 |  |  | 6190 | -0.084 | -0.4248 | No |
| 123 | LZTS1 |  |  | 6222 | -0.086 | -0.4271 | No |
| 124 | EHBP1 |  |  | 6312 | -0.091 | -0.4359 | No |
| 125 | DNM2 |  |  | 6349 | -0.093 | -0.4387 | No |
| 126 | SMAD5 |  |  | 6355 | -0.093 | -0.4379 | No |
| 127 | RIOK3 |  |  | 6373 | -0.094 | -0.4384 | No |
| 128 | METAP2 |  |  | 6375 | -0.094 | -0.4372 | No |
| 129 | RGS16 |  |  | 6543 | -0.104 | -0.4547 | No |
| 130 | SIPA1L2 |  |  | 6567 | -0.106 | -0.4558 | No |
| 131 | GPN1 |  |  | 6673 | -0.112 | -0.4661 | No |
| 132 | PEX19 |  |  | 6808 | -0.122 | -0.4796 | No |
| 133 | TEAD2 |  |  | 6838 | -0.124 | -0.4811 | No |
| 134 | NEDD4L |  |  | 6876 | -0.128 | -0.4835 | No |
| 135 | ACIN1 |  |  | 6890 | -0.128 | -0.4831 | No |
| 136 | ZEB1 |  |  | 6944 | -0.132 | -0.4872 | No |
| 137 | LINC00667 |  |  | 6988 | -0.136 | -0.4901 | No |
| 138 | MED10 |  |  | 6995 | -0.136 | -0.4888 | No |
| 139 | BCCIP |  |  | 7009 | -0.138 | -0.4882 | No |
| 140 | VEZF1 |  |  | 7040 | -0.140 | -0.4896 | No |
| 141 | UBAP2L |  |  | 7180 | -0.154 | -0.5032 | No |
| 142 | ACLY |  |  | 7208 | -0.156 | -0.5040 | No |
| 143 | NSMCE1 |  |  | 7231 | -0.159 | -0.5042 | No |
| 144 | EIF3E |  |  | 7253 | -0.162 | -0.5042 | No |
| 145 | CSNK1A1 |  |  | 7260 | -0.162 | -0.5025 | No |
| 146 | ZNF146 |  |  | 7359 | -0.176 | -0.5111 | No |
| 147 | SCRN1 |  |  | 7434 | -0.185 | -0.5169 | No |
| 148 | LHX2 |  |  | 7455 | -0.187 | -0.5164 | No |
| 149 | NFE2L2 |  |  | 7462 | -0.187 | -0.5143 | No |
| 150 | CCNB1IP1 |  |  | 7481 | -0.189 | -0.5136 | No |
| 151 | NONO |  |  | 7563 | -0.199 | -0.5199 | No |
| 152 | GLUL |  |  | 7594 | -0.203 | -0.5204 | No |
| 153 | SALL1 |  |  | 7597 | -0.204 | -0.5176 | No |
| 154 | CDK2AP1 |  |  | 7630 | -0.208 | -0.5182 | No |
| 155 | ETF1 |  |  | 7748 | -0.225 | -0.5283 | Yes |
| 156 | PHB |  |  | 7753 | -0.226 | -0.5254 | Yes |
| 157 | FBL |  |  | 7780 | -0.230 | -0.5250 | Yes |
| 158 | GDI2 |  |  | 7806 | -0.235 | -0.5244 | Yes |
| 159 | EIF3A |  |  | 7813 | -0.236 | -0.5216 | Yes |
| 160 | FDFT1 |  |  | 7852 | -0.244 | -0.5224 | Yes |
| 161 | MMADHC |  |  | 7911 | -0.257 | -0.5252 | Yes |
| 162 | ZC3H15 |  |  | 7947 | -0.266 | -0.5253 | Yes |
| 163 | ENY2 |  |  | 7951 | -0.266 | -0.5217 | Yes |
| 164 | MAD2L2 |  |  | 7998 | -0.275 | -0.5229 | Yes |
| 165 | PSMA3 |  |  | 8006 | -0.277 | -0.5197 | Yes |
| 166 | EZR |  |  | 8030 | -0.281 | -0.5182 | Yes |
| 167 | NFIX |  |  | 8090 | -0.295 | -0.5206 | Yes |
| 168 | DNAJC8 |  |  | 8117 | -0.300 | -0.5191 | Yes |
| 169 | CCT5 |  |  | 8125 | -0.302 | -0.5155 | Yes |
| 170 | RPS27L |  |  | 8126 | -0.302 | -0.5111 | Yes |
| 171 | C8orf59 |  |  | 8259 | -0.342 | -0.5211 | Yes |
| 172 | ENC1 |  |  | 8274 | -0.346 | -0.5176 | Yes |
| 173 | C19orf70 |  |  | 8307 | -0.356 | -0.5160 | Yes |
| 174 | GLO1 |  |  | 8338 | -0.364 | -0.5141 | Yes |
| 175 | LDHB |  |  | 8372 | -0.379 | -0.5123 | Yes |
| 176 | NAP1L1 |  |  | 8404 | -0.389 | -0.5101 | Yes |
| 177 | HNRNPC |  |  | 8413 | -0.392 | -0.5053 | Yes |
| 178 | EEF1D |  |  | 8455 | -0.412 | -0.5039 | Yes |
| 179 | TMEM158 |  |  | 8499 | -0.434 | -0.5025 | Yes |
| 180 | CHMP2A |  |  | 8561 | -0.464 | -0.5026 | Yes |
| 181 | HNRNPA1 |  |  | 8570 | -0.469 | -0.4966 | Yes |
| 182 | PRDX1 |  |  | 8584 | -0.476 | -0.4911 | Yes |
| 183 | HMGN2 |  |  | 8608 | -0.496 | -0.4865 | Yes |
| 184 | MARCKS |  |  | 8667 | -0.537 | -0.4852 | Yes |
| 185 | C12orf57 |  |  | 8678 | -0.545 | -0.4783 | Yes |
| 186 | SCAND1 |  |  | 8689 | -0.550 | -0.4714 | Yes |
| 187 | NPM1 |  |  | 8706 | -0.566 | -0.4649 | Yes |
| 188 | HIGD2A |  |  | 8708 | -0.569 | -0.4567 | Yes |
| 189 | RPL13A |  |  | 8714 | -0.578 | -0.4487 | Yes |
| 190 | POLR2L |  |  | 8715 | -0.578 | -0.4402 | Yes |
| 191 | PDCD5 |  |  | 8729 | -0.592 | -0.4330 | Yes |
| 192 | SKP1 |  |  | 8756 | -0.617 | -0.4269 | Yes |
| 193 | COMMD6 |  |  | 8772 | -0.628 | -0.4194 | Yes |
| 194 | RPL23 |  |  | 8781 | -0.636 | -0.4110 | Yes |
| 195 | GSTP1 |  |  | 8795 | -0.647 | -0.4030 | Yes |
| 196 | RPL21 |  |  | 8802 | -0.655 | -0.3940 | Yes |
| 197 | FABP5 |  |  | 8829 | -0.684 | -0.3870 | Yes |
| 198 | RPS24 |  |  | 8832 | -0.688 | -0.3771 | Yes |
| 199 | RPL10 |  |  | 8835 | -0.690 | -0.3672 | Yes |
| 200 | H3F3B |  |  | 8846 | -0.705 | -0.3579 | Yes |
| 201 | SUMO2 |  |  | 8852 | -0.712 | -0.3480 | Yes |
| 202 | RPS27 |  |  | 8865 | -0.727 | -0.3387 | Yes |
| 203 | RPS27A |  |  | 8875 | -0.742 | -0.3289 | Yes |
| 204 | RPL35A |  |  | 8903 | -0.777 | -0.3205 | Yes |
| 205 | RPLP0 |  |  | 8904 | -0.777 | -0.3091 | Yes |
| 206 | RPL18 |  |  | 8907 | -0.780 | -0.2979 | Yes |
| 207 | RPS25 |  |  | 8909 | -0.787 | -0.2864 | Yes |
| 208 | BTF3 |  |  | 8911 | -0.789 | -0.2749 | Yes |
| 209 | ACTB |  |  | 8912 | -0.793 | -0.2633 | Yes |
| 210 | RPL3 |  |  | 8916 | -0.803 | -0.2518 | Yes |
| 211 | RPS16 |  |  | 8917 | -0.812 | -0.2399 | Yes |
| 212 | RPS18 |  |  | 8926 | -0.829 | -0.2286 | Yes |
| 213 | RPS6 |  |  | 8929 | -0.837 | -0.2166 | Yes |
| 214 | RPL36 |  |  | 8931 | -0.843 | -0.2043 | Yes |
| 215 | RPLP2 |  |  | 8932 | -0.844 | -0.1919 | Yes |
| 216 | RPS19 |  |  | 8941 | -0.855 | -0.1802 | Yes |
| 217 | RPL13 |  |  | 8944 | -0.862 | -0.1678 | Yes |
| 218 | RPL12 |  |  | 8945 | -0.864 | -0.1551 | Yes |
| 219 | RPS5 |  |  | 8946 | -0.865 | -0.1424 | Yes |
| 220 | RPL4 |  |  | 8950 | -0.881 | -0.1298 | Yes |
| 221 | PKM |  |  | 8959 | -0.915 | -0.1172 | Yes |
| 222 | RPLP1 |  |  | 8968 | -0.958 | -0.1041 | Yes |
| 223 | RPS15 |  |  | 8970 | -0.965 | -0.0900 | Yes |
| 224 | RPL8 |  |  | 8971 | -0.992 | -0.0754 | Yes |
| 225 | RPL28 |  |  | 8972 | -0.993 | -0.0609 | Yes |
| 226 | RPSA |  |  | 8973 | -1.005 | -0.0461 | Yes |
| 227 | RPL41 |  |  | 8974 | -1.013 | -0.0312 | Yes |
| 228 | RPS8 |  |  | 8976 | -1.044 | -0.0160 | Yes |
| 229 | TMSB4X |  |  | 8983 | -1.142 | 0.0001 | Yes |
Table: GSEA details [plain text format]

  

Fig 2: RICHARDS\_NOWAKOWSKI\_SCIENCE\_2017\_IPC\_NEN3\_UPREG: Random ES distribution      
 Gene set null distribution of ES for **RICHARDS\_NOWAKOWSKI\_SCIENCE\_2017\_IPC\_NEN3\_UPREG**
